# Toll-Like Receptor Transcriptome in the HPV-Positive Cervical Cancer Microenvironment

**DOI:** 10.1155/2012/785825

**Published:** 2011-10-13

**Authors:** Correne A. DeCarlo, Bruce Rosa, Robert Jackson, Sarah Niccoli, Nicholas G. Escott, Ingeborg Zehbe

**Affiliations:** ^1^Probe Development and Biomarker Exploration Thunder Bay Regional Research Institute, Thunder Bay, ON, Canada P7B 6V4; ^2^Biorefining Research Initiative and Department of Biology, Lakehead University, Thunder Bay, ON, Canada P7B 5E1; ^3^Thunder Bay Regional Health Sciences Centre, Department of Pathology, Thunder Bay, ON, Canada P7B 6V4

## Abstract

The human papillomavirus (HPV) directly infects cervical keratinocytes and interferes with TLR signalling. To shed light on the effect of HPV on upstream receptors, we evaluated TLRs 1–9 gene expression in HPV-negative normal and HPV-positive pre-malignant and malignant *ex vivo* cervical tissue. Quantitative real-time polymerase chain reaction was performed separately for epithelial and stromal tissue compartments. Differences in gene expression were analyzed by the Jonckheere-Terpstra trend test or the Student's *t*-test for pairwise comparison. Laser capture microdissection revealed an increase in TLR3 and a decrease in TLR1 mRNA levels in dysplastic and carcinoma epithelium, respectively. In the stroma, a trend of increasing TLR 1, 2, 5, 6, and 9 mRNA levels with disease severity was found. These findings implicate the involvement of TLR3 and TLR1 in early and late cervical carcinogenesis, respectively, suggesting that stromal upregulation of TLRs may play a role in cervical disease progression.

## 1. Introduction

The most important role of toll-like receptors (TLRs) in host defence is the regulation of innate and adaptive immune responses by epithelial cells, the first line of protection at, for example, the respiratory, gastrointestinal tract, skin and genitourinary mucosal sites. Present on a variety of cell types, TLRs play an essential role in innate immune system function by recognizing discrete exogenous pathogen-associated molecular patterns (PAMPs) and endogenous damage-associated molecular pattern (DAMP) ligands to induce an innate immune response [1]. Ten functional TLRs have been identified and characterized in humans. Among these, TLRs 3, 4, 7, 8, and 9 have been found to play a critical role in antiviral immunity by triggering the downstream production of interferons (IFNs) including IFN-*α*, IFN-*β* and IFN-*γ* [1, 2]. IFNs are important components of the innate immune response against invading pathogens. They are synthesized during initial infection and evoke antiviral, antitumour, and immune-regulatory activity which gives protection to surrounding cells [3–5]. The IFN group is split into two classes; Type I IFNs encompass IFN-*α*, -*β*, -*κ*, -*δ*, -*ε*, -*ω*, and -*τ*  [6], and generate antiviral activity [7], while IFN-*γ* is the only type II IFN [7] and is involved in the regulation of immune and inflammatory responses [8]. IFN-*κ* is a newly identified type I IFN and is known to have similar antiviral properties. However, it is distinct from other type I IFNs as it signals in a discrete autocrine, rather than paracrine manner [9].

TLRs 3, 7, 8, and 9 can be activated by viral products such as double stranded RNA [10], single stranded RNA [11], and double stranded CpG-rich DNA [12], respectively, whereas cell-surface TLR4 is stimulated by lipopolysaccharide (LPS) and some viral proteins [13]. TLRs 1 and 6 recognize bacterial and mycoplasma lipoproteins, respectively [14–16] while acting in cooperation with TLR2, whereas TLR5 is specifically stimulated by bacterial flagellin [17, 18]. In addition, damage-associated molecular patterns (DAMPS) (which include heat shock proteins, high-mobility group box 1 (HMGB1), uric acid crystals, hyaluronan, heparin sulfate, messenger RNA, surfactant protein A, and various products of the extracellular matrix such as fibronectin and fibrinogen) have been suggested to activate TLRs [1]. 

The critical function of TLRs in innate immune functioning is well studied, but, in recent years, their role in tumour genesis and cancer progression has become an active field of research. Because of their innate immunity signalling, TLRs play an important gate keeper role in controlling the production of proinflammatory cytokines and chemokines as well as IFNs. It has recently been speculated that aberrant TLR expression and signalling in cancer cells and the resulting downstream cascades can directly promote tumour progression [19]. Indeed, aberrant TLR1, 2, 3, 4, 5, 6, and 9 signalling has been implicated in a variety of human cancers [1]. 

Cervical cancer is the second most common malignancy in women worldwide with human papillomavirus (HPV) recognized as its causative agent [20]. HPV is a double stranded DNA virus which infects the squamous epithelium of the uterine cervix. Most infections are cleared by the host, but a proportion of women are unable to clear the infection, resulting in cervical lesions. Although persistent HPV infection is necessary, it is insufficient for the development of cervical cancer. HPV can directly inhibit the function of TLR downstream molecules involved in the IFN pathway [21, 22] and accumulating evidence supports an interaction between TLRs and HPV [23–25]. TLR expression throughout the course of cervical carcinogenesis has not been addressed previously and requires further exploration taking the entire tumour microenvironment into consideration. In this regard, we have recently characterized cytokine and IFN gene expression levels in the normal, premalignant, and malignant epithelium and surrounding stroma of *ex vivo* cervical tissue by laser capture microscopy and quantitative real-time polymerase chain reaction [26]. Compared to normal tissue, these molecules were lacking or showed low expression in the diseased epithelium while all of them were aberrantly increased in the tumour stroma. To provide a complete picture of innate immune responses in the same tissue samples, here we report a comprehensive transcriptome analysis of all upstream TLRs involved in these downstream pathway disturbances.

## 2. Materials and Methods

### 2.1. Sample Preparation

Cervical biopsies were obtained with written consent from women attending the Colposcopy Clinic at the Thunder Bay Regional Health Sciences Centre (TBRHSC) between November 2005 and November 2006. The study has been approved by the local Research Ethics Team at the TBRHSC (no. 21.05). Biopsies were snap frozen in liquid nitrogen and immediately transferred to −80°C. Tissue was sectioned on a cryostat (Leica CM1850, Leica Microsystems, Richmond Hill, ON, Canada), maintaining tissue temperature at −20°C using Tissue Tek embedding medium (O.C.T. Compound, Sakura Finetek, Torrance, Calif, USA) and sectioned. For laser capture microdissection (LCM) analysis, 8 *μ*m-thick tissue sections were cut, adhered to uncharged slides, and kept in −20°C prior to LCM preparation. All tweezers, brushes, and surfaces were cleaned with DEPC-treated 70% (v/v) ethanol between specimens to reduce RNAse activity and RNA cross-contamination. 

All samples collected in this study were thoroughly tested for RNA integrity, as previously described [26, 27]. Of 110 samples, 25 had enough remaining tissue suitable for LCM analysis. The 25 samples utilized in this study along with their HPV infection status are listed in Table 1. In total, 11 of 25 samples were negative for HPV and diagnosed as morphologically normal. Of the 14 diseased samples, 4 were diagnosed as low-grade lesions (LSIL), 6 were high-grade lesions (HSIL), and 4 were invasive carcinomas. Importantly, all normal tissue was negative for HPV infection, and all diseased tissue was positive for HPV infection. Histological diagnosis of the snap-frozen biopsy was verified before and after LCM and correlated with the diagnosis of the biopsy that was taken for clinical purposes.

### 2.2. HPV Typing and Specimen Classification

Ten tissue sections were cut and then taken immediately for LCM analysis, and placed into 1.5 mL microcentrifuge tubes for DNA extraction. DNA extraction was performed using the Qiagen QIAamp DNA micro kit (Qiagen, Mississauga, ON, Canada) following the manufacturer's protocol for tissue samples. Samples were HPV genotyped using Luminex hybridization at the National Microbiology Laboratory (Winnipeg, B, Canada), as previously described [26]. In addition, PCR analysis [38] and p16-based testing using the CINtec p16 immunocytochemistry staining kit (CINtec p16INK4a Cytology Kit; mtm laboratories, Westborough, Mass, USA) were performed to visualize HPV infection. For histopathological diagnosis, sections were cut and processed for hematoxylin and eosin staining [39] and diagnosed by the same pathologist (N.E.). Tissue samples were diagnosed as morphologically normal, low/high-grade lesion, and cervical carcinoma tissue. For this study, samples were categorized as HPV-negative and morphologically normal (Normal; *n* = 11), HPV-positive and morphologically dysplastic (Dysplasia; *n* = 10) and HPV-positive and morphologically malignant (Carcinoma; *n* = 4).

### 2.3. RNA Extraction and Integrity Assessment

RNA was extracted from 10 × 10 *μ*m thick tissue sections of microdissected cervical cells or keratinocytes as previously described [27]. The quality and quantity of RNA extracted from cervical tissue was assessed using the Bio-Rad Experion automated electrophoresis system. RNA integrity from samples was assessed as previously described [27].

### 2.4. Laser Capture Microdissection

Tissue specimens were prepared for laser capture microdissection (LCM) using the Arcturus Histogene Frozen Section Staining Kit, as previously described [27]. Up to five thousand captures (~25,000 cells) of both epithelium and stroma for each tissue specimen were taken. Macrocaps were cleaned of unwanted debris using CapSure pads (Arcturus) prior to being deposited into 0.5 mL microfuge tubes for RNA extraction. The laser spot size was consistently 15 *μ*m in diameter whereas the laser power and duration ranged from 80 to 95 mW and 0.65 to 0.8 millisecond duration between specimens. Images of the excision process have been previously reported [27].

### 2.5. Quantitative Real-Time Polymerase Chain Reaction

RNA isolated from samples was reverse transcribed to complementary DNA (cDNA) using the High Capacity cDNA Archive Kit (Applied Biosystems) according to manufacturer's directions with random hexamer primers. RNA isolated from microdissected samples was reverse transcribed at a minimum of 0.6 ng/*μ*L (ranging from 0.6–2.8 ng/*μ*L), depending on acquired LCM sample RNA concentration. Complementary DNA (cDNA) was amplified using the TaqMan PreAmp Master Mix Kit (Applied Biosystems). Gene amplification uniformity was assessed as previously described [27]. Reactions were carried out according to the quantitative real-time polymerase chain reaction (qRT-PCR) protocol specified in the TaqMan PreAmp Master Mix Kit. Triplicate reactions of 25 *μ*L volume were added to a 96-optical well plate (Applied Biosystems) and incubated at standard qRT-PCR conditions (50°C for 5 minutes 95°C for 10 minutes and then cycled at 95°C for 15 seconds and 60°C for 1 minute for 40 cycles (detection limit)). qRT-PCR was repeated for 10% of *ex vivo* human samples to ensure result reliability. Target genes were normalized to the housekeeping gene hypoxanthine phosphoribosyltransferase1 (HPRT1), as previous results indicate its expression is unaffected by HPV infection [27]. TaqMan gene expression on demand assays for HPRT1 and TLRs 1 through 9 were used (Applied Biosystems). Negative controls where cDNA was omitted or the enzyme was missing in the RT reaction were run to monitor for contamination or nonspecific primer binding. A positive control was included on every plate to control for variation between runs. Relative quantification of target genes was performed using auto Ct and baseline settings and a threshold of 0.20 (Applied Biosystems 7300/7500/7500 Fast Real-Time PCR System Software).

### 2.6. Statistical Analysis

The difference in cycle thresholds (ΔCT) between HPRT1 and the TLRs was calculated for each gene in each biological sample. These values were converted to relative expression values using 2^−ΔCT^ [40]. Statistical differences in gene expression levels were assessed using the Jonckheere-Terpstra [41] test for trends when analyzing the three tissue groups together and using the Student's *t*-test for pairwise comparison when comparing diseased versus normal tissue. *P*-values for all statistical comparisons are reported with at most two significant digits. Statistical significance is stated when *P* ≤ 0.05. All tests are two sided if not otherwise indicated and based on the “exact” version. Calculations were performed using Microsoft Excel version 12 (2008) and Cytel Studio StatXact7 version 7.0.0 (2005), Cytel Software Corporation (http://www.cytel.com/) and the SAS statistical analysis package (Version 9.1.2, http://support.sas.com/) for statistical tests.

## 3. Results

### 3.1. Baseline TLR mRNA Levels in Normal, HPV-Negative Ex Vivo Cervical Tissue

**Figure 1:**
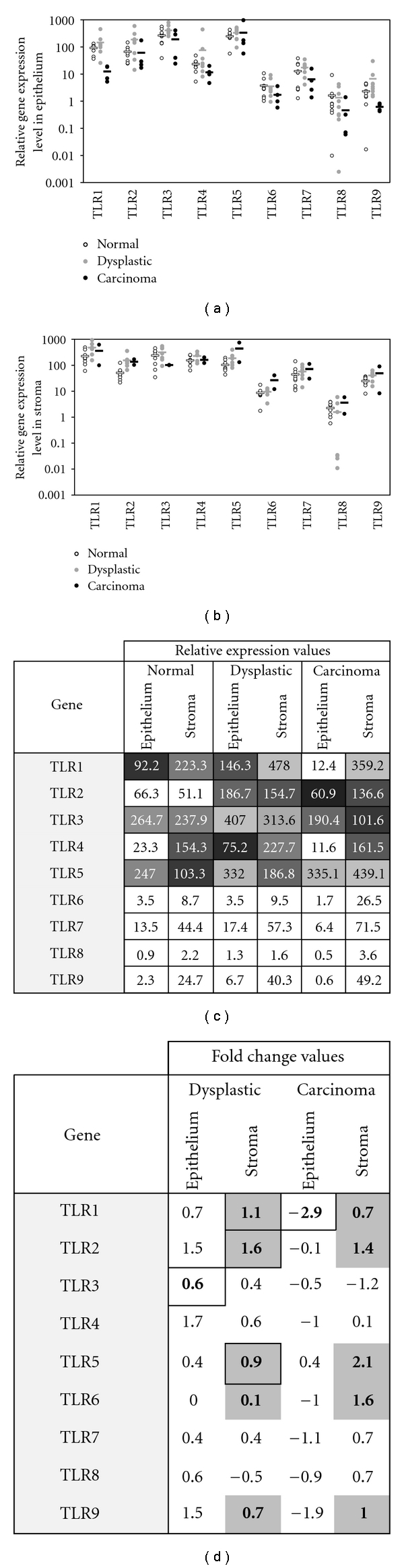
TLR gene expression in normal, dysplastic and carcinoma *ex vivo* cervical tissue. Scatterplot indicating relative TLR gene expression levels (logged) in normal, dysplastic and carcinoma samples in the epithelium (a) and stroma (b) compartment. Horizontal bars show the average expression levels per group for each gene. Asterisks show significant expression differences from normal samples according to the Student's *t*-test for pairwise comparisons. Reported average relative expression values for each gene in both tissue compartments (c), values calculated according to 2^−ΔCT^ ∗ 1,000. Fold change values for TLRs in dysplastic and carcinoma samples (d). Significant fold change values according to the Student's *t*-test for pairwise comparison of two types are indicated with a thick border, and genes with significant upregulation trends according to the Jonckheere-Terpstra test are shaded gray.

To define baseline mRNA levels of TLRs in healthy cervical tissue, 11 cervical biopsies from histologically normal, HPV-negative women were analyzed, as shown in Table 1. Gene expression levels in the epithelium and stroma of healthy samples are shown in Figures 1(a) and 1(b), respectively. High levels of TLRs 1, 2, 3, and 5 mRNA were found in relation to lower levels of TLRs 4, 6, 7, and 9, with TLR 8 found at the lowest levels in normal epithelium. Alternatively, high levels of TLRs 1, 3, 4 and 5 mRNA were found in relation to lower levels of TLRs 2, 6, 7 and 9, with TLR 8 again found at the lowest levels in normal stroma. Importantly, mRNA was found for all TLRs in healthy cervical epithelium and stroma.

### 3.2. TLR3 Increases in Dysplasia Epithelium While TLR1 Decreases in Carcinoma Epithelium

To characterize TLR mRNA levels in dysplastic and carcinoma epithelium, 14 HPV-positive, diseased cervical epithelial samples were analyzed, as shown in Table 1. Previous studies have demonstrated an interaction between TLR signalling and HPV [25] upstream of the IFN pathway. HPV infections disrupt cytokine expression, and the E6 and E7 oncoproteins particularly target the type I IFN pathway [42]. We previously reported IFN-*κ* and IL-10 downregulation in dysplastic and carcinoma epithelium [26]. We therefore hypothesized that TLR mRNA expression levels would be altered due to the presence of HPV in the epithelium, to possibly reflect these downstream findings in the stroma. Using LCM to isolate the epithelium in whole cervical biopsy sections, the current analysis revealed several significant differences in TLR mRNA levels in HPV-positive, diseased compared to healthy, HPV-negative epithelium (Figure 1(a)). Importantly, all epithelial samples were positive for TLR expression (Figure 1(c)). While TLR3 levels were found to be significantly higher in dysplastic epithelial samples (*P* = 0.05, Student's *t*-test; Figure 1(d)), TLR1 levels were found to be significantly lower in carcinoma epithelium (*P* = 0.01, Student's *t*-test; Figure 1(d)). A trend analysis did not reveal any further differences in TLR expression levels in the epithelium. Therefore, the most striking differences found in the epithelium were the increased TLR3 gene expression levels in dysplastic epithelium and the decreased TLR1 gene expression levels in carcinoma epithelium; the latter of which corresponds to decreased IFN-*κ* levels previously reported in the same samples [26].

### 3.3. TLRs 1, 2, 6 as well as 5 and 9 Increase with Disease Severity in Cervical Stroma

To characterize TLR mRNA levels in dysplastic and carcinoma stroma, 14 HPV-positive, diseased cervical stromal samples were analyzed, as shown in Table 1. Previous LCM analysis of the same tissue samples revealed increased downstream IFN mRNA levels in dysplastic and carcinoma stroma [26]. TLRs are upstream elicitors of the IFN pathways, and we therefore expected to see TLR alterations in the stroma with cervical disease progression. Using LCM to isolate the stroma in whole cervical biopsy sections, the current analysis revealed several significant differences in TLR mRNA levels in HPV-positive, diseased compared to healthy, HPV-negative stroma (Figure 1(b)). Importantly, all stromal samples were positive for TLR expression (Figure 1(c)). A trend analysis revealed increasing expression trends with disease severity in diseased stroma for TLRs 1, 2, 6 as well as 5 and 9 (*P* = 0.05, 0.001, 0.02, 0.01, and 0.04 resp., Jonckheere-Terpstra test; Figure 1(d)). Although a trend exists, strong increases in TLR 1, 2, and 5 mRNA levels were found predominately in dysplastic stroma (*P* = 0.02, 0.001 and 0.05 resp., Student's *t*-test). Thus, the most striking results found in the stroma compartment were the increased mRNA levels for five TLRs, which coincides with our previous reported increase in downstream IL-10, IFN-*κ*, IFN-*β*, and IFN-*γ* in the stroma of the same samples [26].

## 4. Discussion

Through the *ex vivo* analysis of gene expression in HPV-negative, healthy as well as HPV-positive premalignant and malignant cervical tissue, we report a transcriptome model with an emphasis on tissue compartmentalization for innate immune signalling pathways in cervical carcinogenesis. By conducting a systematic literature survey (Table 2), we did not find a comparable study which elucidated the transcriptome of TLRs (current study) and downstream effectors [26] in cervical carcinogenesis using LCM and qPCR. Only one transcriptome study of cervical cancer development utilizing LCM to separate tissue compartments was found [43]. However, differential expression of TLR or IFN expression was not discovered in that investigation [43], likely due to low transcription levels undetectable using microarray analysis. Other transcriptome studies (Table 2) used primary human keratinocytes and/or cervical cancer-derived cell lines [33, 35], whole biopsies [24, 28–31, 34, 36] or both [25, 32, 37] or cytobrush samples [23, 44], but to date none have analyzed the transcriptome in isolated tissue components. Here, we found all TLRs to be expressed *ex vivo* by healthy, HPV-negative cervical keratinocytes and stroma, albeit at different levels: mRNA for TLRs 1, 3 and 5 tended to be most abundant compared to TLRs 6 to 9, especially TLR8, which were expressed at consistently lower levels in either tissue compartment (Figures 1(a) and 1(b)), while TLR 2 was expressed highest in epithelium, and TLR4 was expressed highest in stroma. These findings are partly concordant with a previous investigation [35], where TLRs 1, 2, 3, 5, and 6 were found most abundant and TLRs 4, 7, 8, s and 9 were expressed at lower levels in normal ectocervical keratinocytes. Discordant TLR 6 findings may be a result of keratinocytes being analyzed within their natural tissue context, complete with signals from the underlying dermal stroma, in the current analysis.

Compared to normal tissue, TLR3 was found to be higher expressed in premalignant epithelium and TLR1 to be expressed lower in malignant epithelium. Similar to previous findings, [24, 25] we found a trend for TLRs 4 and 9 to be decreased in carcinoma epithelium, although not significantly. Likewise, other TLR receptors were also decreased, with the exception of TLR8, in the carcinoma epithelium, but statistical significance was not reached, possibly due to individual differences in expression levels in *ex vivo* samples. This decreasing trend in TLR expression coincides with our previous finding that IFN-*κ* was abolished in diseased epithelium, [26] and other studies have similarly revealed lower levels of type I and II IFNs in cervical malignancy, [30, 36, 37] although the isolation of cervical epithelium was not performed in these studies. In contrast to low TLR levels in carcinoma epithelium, TLRs 1, 2, 6, as well as 5 and 9 showed higher expression in premalignant and malignant stroma compared to normal tissue. These results coincide with previously reported increases in IL-10, IFN-*κ*, IFN-*β*, and IFN-*γ* mRNA levels as well as infiltrating monocytes and dendritic cells in the cervical stroma in these samples [26]. Previous investigations have also revealed elevated mRNA level of TLRs 5 and 9 in cervical carcinoma tissue, [29, 34] and in contrast, higher IFN-*γ* and IL-10 have been associated with lower incidences of cervical dysplasia, [28] however neither investigation entailed tissue microdissection to reveal the cell types responsible.

**Figure 2:**
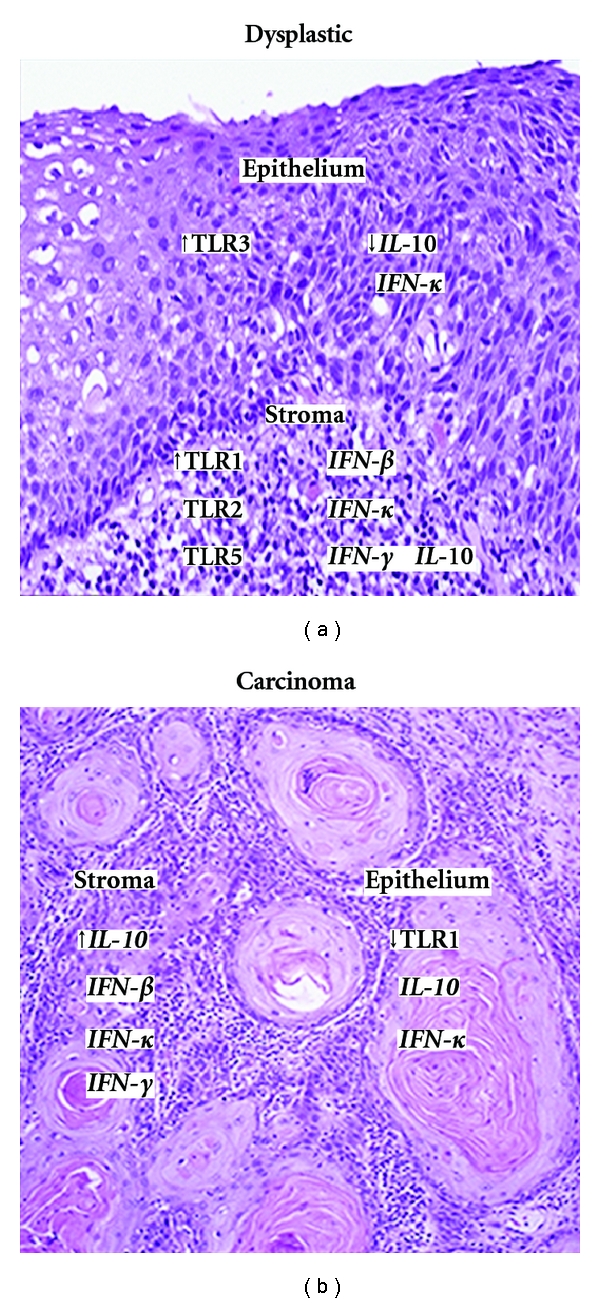
Overall gene expression models for ex vivo dysplastic (a) and carcinoma (b) cervical epithelium and stroma. Genes presented from the current analysis as either up or down-regulated compared to HPV-negative, normal cervical tissue. Italicized genes are based on data we reported previously [19]. In addition to the data presented, trend analyses also revealed a significant up-regulation of TLRs 1, 2, 5, 6, and 9 in the stroma with disease severity.

Based on our combined gene expression findings, we propose a disease scenario in the cervical microenvironment (Figure 2). During early cervical carcinogenesis, when the cervical epithelium is still undergoing differentiation, which typically takes place parallel to the viral life cycle of HPV, TLR3, a classical receptor eliciting antiviral responses via IFN-regulatory factor (IRF) 3, expression increases in dysplastic epithelium. This upregulation may be an attempt by the infected cells to initiate immune responses against the HPV infection [25]. The concomitant downregulation of downstream IFN-*κ* in premalignant epithelium samples could likely be attributed to interference of HPV E6 with the IFN pathway by blocking IRF 3 [45]. We also show that TLR1 was significantly decreased in carcinoma epithelium and may represent the sole upstream TLR significantly affected in keratinocytes in late carcinogenesis. With disease severity, the presence of HPV in the epithelium may lead to higher expression of TLRs in diseased stroma, which can account for the increases in IFNs previously found [26]. Altogether, these data demonstrate that the TLR pathways are not being induced in late carcinogenesis in the cervical epithelium, yet the presence of HPV indirectly triggers TLR pathway activation in the cervical stroma, eliciting downstream innate immune responses. However, IFNs may play a direct role in the expression levels of TLRs, as recent evidence points towards an intriguing mechanism by which type I IFNs can directly induce TLRs 1, 3, 5 and 7, [46] which are some of the TLRs found to be up-regulated in diseased cervical stroma in the present study. The presence of up-regulated responses in the stroma and not in the epithelium may contribute to the persistence of HPV and ultimately cultivate the progression of the lesion.

 Several mechanisms have been suggested for IFN-*κ* downregulation in malignant epithelium: (i) downregulation of TLR9 by HPV16 E6, [25] (ii) methylation of the IFN-*κ* promoter [32] and (iii) downregulation of IFN-*κ* by the HPV16 E6 oncoprotein [47]. The present findings suggest yet another potential underlying mechanism which is linked to the three-fold and statistically significant decreased expression level of TLR1 in malignant epithelium. In contrast, the increased TLR1 expression in malignant stroma is likely due to increased monocyte infiltration, implicating DAMPS from dying cancer cells as potential ligands. This notion requires further investigation because TLR1 is usually involved in the activation of downstream proinflammatory cytokines rather than IFNs. However, recent research suggests that in addition to the canonical TLRs 3, 4, 7, 8, and 9, the TLR2 subfamily (TLR1, 2 and 6) is associated with IFN responses [48]. 

HPV-associated cancers are a worldwide health concern, and since the recently approved prophylactic vaccine against common high-srisk HPV types is not a treatment option, there is a great need for studies to characterize the effect of HPV on innate immune responses to advance treatment options. Because of their wide-ranging impact upon both innate and adaptive immunity in several disease settings, TLRs and their signalling pathways emerge as attractive therapeutic targets [49]. Based on our present findings using a highly defined sample size, TLRs 3 and 1 could be further investigated for diagnostic and eventually prognostic marker use. Although TLRs can mediate host innate immune system signalling, overexpression of TLRs has been paradoxically found in many tumour cases [50]. In line with our findings in diseased cervical stroma, TLR antagonists, for example, which act to block MyD-88, could confer therapeutic benefit in cervical cancer patients.

##  Conflicts of Interest

The authors declare that they have no conflicts of interest.

## Figures and Tables

**Table 1:** Sample size. Twenty-five frozen cervical biopsies were categorized into 3 groups; normal tissue, case IDs N1–N11; dysplastic tissue, case IDs D1–D10; invasive carcinoma tissue, case IDs C1–C4. Laser capture microdissection was used to separate each specimen into epithelium and stroma compartments. The HPV infection status is shown, and the specific HPV type is noted when known.

Case ID	Morphological diagnosis	HPV infection status	Case ID	Morphological diagnosis	HPV infection status
N1	Normal	Negative	D3	LSIL	Types 66, 82
N2	Normal	Negative	D4	LSIL	Types 6, 66
N3	Normal	Negative	D5	HSIL	Type 16
N4	Normal	Negative	D6	HSIL	Type 16
N5	Normal	Negative	D7	HSIL	Type 33
N6	Normal	Negative	D8	HSIL	Type 16
N7	Normal	Negative	D9	HSII	Type 16
N8	Normal	Negative	D10	HSII	TYPe 39
N9	Normal	Negative	C1	Carcinoma	Type 16
N10	Normal	Negative	C2	Carcinoma	HR HPV+
N11	Normal	Negative	C3	Carcinoma	HR HPV+
D1	LSIL	Type 31	C4	Carcinoma	HR HPV+
D2	LSIL	Type 51			

LSIL: low-grade squamous intraepithelial lesion; HR: high risk; HSIL: high-grade squamous intraepithelial lesion.

**Table 2:** Summary of literature of dysregulated innate immunity genes in cervical tissue. A systematic literature search for investigations into TLR and IFN expression in HPV-infected cervical tissue or cell lines was performed using the following search terms in the PubMed database: “Cervical” and “interferon gamma” (489 hits) or “interferon beta” (202 hits) or “interferon kappa” (3 hits) or “toll-like receptor” (59 hits). Acronyms IFN-*γ*, -*β* and -*κ* as well as TLR in combination with “cervical” were also tried. The listed citations are articles published up to 5 January 2011 which describe TLR and IFN gene regulation and dysregulation. Only articles published in English were considered, and only articles relevant for our study were included in the table.

Gene	Material			Sample type	Method	Group
	Normal	CIN	Carcinoma			
	15	11	13	Full biopsy	qPCR	Pao et al. 1995 [28]
	4	12	N/S	Full biopsy, HeLa	qPCR, IHC	de Gruijl et al. 1999 [29]
	N/A	N/A	52	Full biopsy	qPCR, IHC	Gey et al. 2003 [30]
IFN-*γ*	10	N/A	29	Full biopsy	qPCR, IHC	Alcocer-González et al. 2006 [31]
	6	N/A	6	Cytobrush	cDNA array	Manavi et al. 2007 [31]
	150	198	N/A	Full biopsy	qPCR	Scott et al. 2009 [32]
	N/S	N/S	N/S	Full biopsy, LCM	qPCR	DeCarlo et al. 2008 [27]
	11	10	4	Full biopsy, LCM	qPCR	DeCarlo et al. 2010 [19]

	N/A	N/A	N/A	CK	cDNA array, qPCR	Nees et al. 2001 [33]
IFN-*β*	N/S	N/S	N/S	Full biopsy, LCM	qPCR	DeCarlo et al. 2008 [27]
	11	10	4	Full biopsy, LCM	qPCR	DeCarlo et al. 2010 [19]

	N/S	N/S	N/S	Full biopsy, LCM	qPCR	DeCarlo et al. 2008 [27]
IFN-*κ*	N/S	10	N/A	Full biopsy, PHFK	qPCR, IHC	Rincon-Orozco et al. 2009 [34]
	11	10	4	Full biopsy, LCM	qPCR	DeCarlo et al. 2010 [19]

	3	4	N/A	CK, PHK, SiHa, CaSki, HeLa, C33A	qPCR	Hasan et al. 2007 [25]
TLR1	12	N/A	N/A	CK, VK, ME180, HeLa	qPCR	Herbst-Kralovetz et al. 2008 [35]
	65	65	N/A	Cytobrush	qPCR	Daud et al. 2010 [24]

	3	4	N/A	CK, PHK, SiHa, CaSki, HeLa, C33A	qPCR	Hasan et al. 2007 [26]
TLR2	12	N/A	N/A	CK, VK, ME180, HeLa	qPCR	Herbst-Kralovetz et al. 2008 [35]
	65	65	N/A	Cytobrush	qPCR	Daud et al. 2010 [24]

	3	4	N/A	CK, PHK, SiHa, CaSki, HeLa, C33A	qPCR, IF	Hasan et al. 2007 [26]
TLR3	12	N/A	N/A	CK, VK, ME180, HeLa	qPCR	Herbst-Kralovetz et al. 2008 [35]
	65	65	N/A	Cytobrush	qPCR	Daud et al. 2010 [24]

	3	4	N/A	CK, PHK, SiHa, CaSki, HeLa, C33A	qPCR	Hasan et al. 2007 [26]
TLR4	12	N/A	N/A	CK, VK, ME180, HeLa	qPCR	Herbst-Kralovetz et al. 2008 [35]
	20	125	49	Full biopsy	qPCR, IHC	Yu et al. 2010 [25]

	3	4	N/A	CK, PHK, SiHa, CaSki, HeLa, C33A	qPCR	Hasan et al. 2007 [19]
TLR5	12	N/A	N/A	CK, VK, ME180, HeLa	qPCR	Herbst-Kralovetz et al. 2008 [35]
	9	22	24	Full biopsy	qPCR	Kim et al. 2008 [36]

	3	4	N/A	CK, PHK, SiHa, CaSki, HeLa, C33A	qPCR	Hasan et al. 2007 [26]
TLR6	12	N/A	N/A	CK, VK, ME180, HeLa	qPCR	Herbst-Kralovetz et al. 2008 [35]
	65	65	N/A	Cytobrush	qPCR	Daud et al. 2010 [24]

	3	4	N/A	CK, PHK, SiHa, CaSki, HeLa, C33A	qPCR	Hasan et al. 2007 [26]
TLR7	12	N/A	N/A	CK, VK, ME180, HeLa	qPCR	Herbst-Kralovetz et al. 2008 [35]
	65	65	N/A	Cytobrush	qPCR	Daud et al. 2010 [24]

	3	4	N/A	CK, PHK, SiHa, CaSki, HeLa, C33A	qPCR	Hasan et al. 2007 [19]
TLR8	12	N/A	N/A	CK, VK, ME180, HeLa	qPCR	Herbst-Kralovetz et al. 2008 [35]
	65	65	N/A	Cytobrush	qPCR	Daud et al. 2010 [24]

	3	4	N/A	Full biopsy, CK, PHK, SiHa, CaSki, HeLa, C33A	qPCR, IF, IHC	Hasan et al. 2007 [26]
	9	22	24	Full biopsy	qPCR	Lee et al. 2007 [37]
TLR9	12	N/A	N/A	CK, VK, ME180, HeLa	qPCR	Herbst-Kralovetz et al. 2008 [35]
	65	65	N/A	Cytobrush	qPCR	Daud et al. 2010 [24]
	20	125	49	Full biopsy	qPCR, IHC	Yu et al. 2010 [25]

CIN, cervical intraepithelial neoplasia; CK, *in vitro* cervical keratinocytes; IF, immunofluorescence; IHC, immunohistochemistry; LCM, laser capture microdissection; N/A, not applicable; N/S, not stated; PHFK, primary human foreskin keratinocytes; PHK, primary human keratinocytes, anatomical origin not specified; qPCR, quantitative real-time polymerase chain reaction; VK, vaginal keratinocytes.
